# Adolescents’ perceptions of tobacco accessibility and smoking norms and attitudes in response to the tobacco point-of-sale display ban in Scotland: results from the DISPLAY Study

**DOI:** 10.1136/tobaccocontrol-2018-054702

**Published:** 2019-05-03

**Authors:** Mirte AG Kuipers, Catherine Best, Michael Wilson, Dorothy Currie, Gozde Ozakinci, Anne-Marie MacKintosh, Martine Stead, Douglas Eadie, Andy MacGregor, Jamie Pearce, Amanda Amos, Sally Haw

**Affiliations:** 1 Department of Public Health, Amsterdam Public Health research institute, Amsterdam UMC, University of Amsterdam, Amsterdam, The Netherlands; 2 Faculty of Health Sciences and Sport, University of Stirling, Stirling, UK; 3 School of Medicine, University of St Andrews, St Andrews, UK; 4 Institute for Social Marketing, Faculty of Health Sciences and Sport, University of Stirling, Stirling, UK; 5 Head of Policy Research, Scotcen Social Research, Edinburgh, UK; 6 Centre for Research on Environment Society and Health, School of GeoSciences, University of Edinburgh, Edinburgh, UK; 7 The Usher Institute of Population Health Sciences and Informatics, College of Medicine and Veterinary Medicine, University of Edinburgh, Edinburgh, UK; 8 Faculty of Health Sciences and Sport, University of Stirlings, Stirling, UK

**Keywords:** smoking, tobacco control, adolescents, young people, point of sale, display, tobacco marketing, social norm, tobacco access

## Abstract

**Background:**

Scotland implemented a ban on open display of tobacco products in supermarkets in April 2013, and small shops in April 2015. This study aimed to quantify changes in perceived tobacco accessibility, smoking norms and smoking attitudes among adolescents in Scotland, following the implementation of partial and comprehensive point-of-sale (POS) tobacco display bans.

**Methods:**

From the Determining the Impact of Smoking Point of Sale Legislation Among Youth (DISPLAY) Study’s 2013–2017 annual surveys we retrieved data comprising 6202 observations on 4836 12–17-year-old adolescents from four schools. Applying generalised estimating equations, associations between time (postban: 2016–2017 vs preban:2013) and three outcomes were estimated. Outcomes were perceived commercial access to tobacco, perceived positive smoking norm (friends think it’s OK to smoke) and positive smoking attitude (you think it’s OK to smoke). Analyses were adjusted for sociodemographics, smoking status, family smoking, friend smoking and e-cigarette use.

**Results:**

Crude trends showed an increase over time in perceived accessibility, norms and attitudes. However, after adjustment for confounders, mainly e-cigarette use, we found significant declines in perceived access (OR = 0.72, 95% CI 0.57 to 0.90) and in positive smoking attitude (OR = 0.67, 95% CI = 0.49 to 0.91), but no change in perceived positive smoking norm (OR = 1.00, 95% CI 0.78 to 1.29). Current/past occasional or regular e-cigarette use was associated with higher odds of perceived access (OR = 3.12, 95% CI 2.32 to 4.21), positive norm (OR = 2.94, 95% CI 2.16 to 4.02) and positive attitude (OR = 3.38, 95% CI 2.35 to 4.87).

**Conclusion:**

Only when taking into account that the use of e-cigarettes increased in 2013–2017 did we find that the POS tobacco display ban in supermarkets and small shops in Scotland was followed by reductions in adolescents’ perceived accessibility of tobacco and positive attitudes towards smoking.

## Introduction

In many countries the tobacco retail environment functions as one of the last forms of legal tobacco advertising,^1^ and in which the tobacco industry heavily invests.^2 3^ Young people are a key target group for these tobacco marketing strategies^4 5^ and studies show that exposure to tobacco displays at the point of sale (POS) increases adolescents’ perceived accessibility of tobacco,^6^ tobacco brand recognition,^7^ estimation of peer smoking,^6^ and smoking susceptibility and initiation.^8 9^ In line with WHO’s Framework Convention on Tobacco Control’s Article 13,^10^ countries are increasingly moving to adopt POS tobacco display bans. It is important to determine the effectiveness of POS display bans in real world settings, to inform other countries on their relative importance for tobacco control in general, and youth smoking prevention in particular.

Scotland introduced a partial POS display ban (ie, in large shops only) in April 2013 and a comprehensive ban, also covering small shops, came into force in April 2015.^11^ Data collected preban showed that 80% of Scottish adolescents reported having seen tobacco displays.^12^ The impact of taking displays out of sight is therefore potentially substantial, but this has not yet been studied.

Although there is some evidence on the impact of POS display bans on adolescent smoking, findings are mixed. Studies evaluating a comprehensive display ban in Ireland and a partial (supermarket) ban in England have not found 12-month effects on smoking prevalence,^13 14^ while studies from Australia and New Zealand, and a European comparative study found a decrease in adolescent smoking in the longer term.^15–17^

In order to understand how smoking may or may not be affected by display bans, we need more insight into the underlying factors through which display bans work. One of the expected short-term effects of reduced exposure to POS displays is a reduction in the perceived ease of access to tobacco products,^18^ which has been found in Norway,^19^ but not in the Europe-wide study.^17^ In England, the proportion of adolescent smokers purchasing cigarettes in shops decreased.^20^ With a decrease in accessibility, smoking is expected to become less acceptable.^18^ Although studies have shown that the perception of whether others smoke decreased,^14 15^ it is unknown whether adolescents’ perceptions of whether others approve of smoking and adolescents’ own attitude towards smoking were affected.

This study aimed to quantify changes in perceived tobacco accessibility and smoking norms and attitudes among young people in Scotland, around the implementation of the partial and comprehensive POS tobacco display bans. Using data from the Determining the Impact of Smoking Point of Sale Legislation Among Youth (DISPLAY) Study, this paper addressed the following research questions:

1. What were the trends in perceived tobacco accessibility and smoking norms and attitudes in Scotland between 2013 (preban) and 2016–2017 (postban)?
2. To what extent did these variables change after the introduction of the partial and comprehensive bans, respectively, compared with before?
3. Were the changes in these variables greater for those adolescents who were more often exposed to tobacco retail environments?

## Methods

### Data

In the DISPLAY Study, annual surveys were conducted among Scottish adolescents in four secondary schools from January to March of 2013–2017. The 2013 survey was therefore conducted before the ban in supermarkets came into force in April 2013, and the 2014 and 2015 surveys were conducted prior to the ban in small shops coming into force in April 2015.

Four medium-sized to large-sized (1100–1200 students), non-denominational schools were selected in the central belt of Scotland. The ethnic minority population in each school was less than 10%, in order to be representative of the majority of large schools in Scotland. Schools were selected to represent higher and lower levels of urbanisation and deprivation. ‘Opt-out’ consent was provided by parents and students. In all five survey years, the participation rate was 86%–87%. More details on the DISPLAY Study are published elsewhere.^18^

For the purpose of this study, we selected all second-year and fourth-year students (approximately 13.5 years and 15.5 years of age, respectively), who were represented in all survey years 2013–2017 (N observations = 7168 and N individuals = 5376). Out of the 5376 individuals, 1791 students were included in two survey waves, as the second-year students were followed up 2 years later as fourth-year students. The data were, therefore, for a third longitudinal and for two-thirds repeat cross-sectional. Out of the 7168 observations, we excluded 269 observations, because at least one of the three outcome variables had a missing value. Six thousand two hundred and two observations of 4836 individuals had complete data on the variables used, and were included in the analysis.

### Measures

#### Time

Time was measured as the survey year (2013–2017). Additionally, time was segmented into year before the partial ban (preban; 2013), years between partial and comprehensive bans (mid-ban; 2014–2015), and years after the comprehensive ban (postban; 2016–2017).

#### Outcome variables

Self-reported perceived tobacco accessibility was measured using the following question: ‘If you, or someone your age, tried to buy cigarettes or tobacco in a shop, do you think you would be successful?’. Response categories included ‘yes’, ‘no’ and ‘don’t know’. ‘Don’t know’ was categorised together with ‘no’, meaning that we distinguished those who were confident that people their age would be able to buy tobacco.

We measured the injunctive social norm (perception of what others think, in short: smoking norm) and the attitude of the individual (short: smoking attitude).^21^ The smoking norm was measured with the question ‘Do your friends think it is OK for people your age to smoke cigarettes or hand-rolled cigarettes (roll-ups)?’. Answer options included ‘they think it’s OK’, ‘they do not think it’s OK’ and ‘don’t know’. Attitude towards smoking was measured as ‘Do you think it is OK for someone your age to do the following? Smoke cigarettes or hand-rolled cigarettes (roll-ups) once a week’, to which students responded ‘it’s OK’, ‘it’s not OK’ or ‘don’t know’. As for accessibility and smoking norm, ‘don’t know’ responses were merged with ‘it’s not OK’ responses.

#### Shop visit frequency

Students reported the frequency per week they visit different types of shops (‘How often, if ever, do you visit (shop type)?’), ranging from every day to never, as used previously.^12^ Students could also opt for ‘don’t know’. Shop types that were likely to sell tobacco were categorised into small shops and large shops, with small shops including: newsagents/corner shops, garage shops/petrol stations, grocery shops or mini marts, fish and chip shops, and other takeaway shops. Supermarkets (excluding supermarket express outlets) were categorised as large shops. For small shops the value of the most visited shop was used as the indicator for shop visit frequency. For both small and large shops a separate variable was computed with three categories: often (every day, most days), sometimes (two or three times a week, about once a week), rarely (less than once a week, never).

#### Covariates

We measured age (in years, 13–17 years), gender (male vs female), ethnicity (non-white vs white), school year (fourth vs second), Family Affluence Scale (FAS), smoking status, smoking by family members and friends, and e-cigarette use. These variables can act as confounders in the trends in outcomes, if their distribution differs between survey years (either due to sample composition or co-occurring trends) and if they affect outcomes.

FAS is a validated scale of material wealth consisting of six items: own bedroom, number of family cars, number of computers, number of family holidays abroad per year, owning a dishwasher and number of bathrooms.^22^ Using principal component analysis, FAS scores were transformed into a single-dimensional score which was then divided into tertiles of high, medium and low FAS.^23^

Smoking status was measured with the question ‘Have you ever smoked cigarettes or hand-rolled cigarettes (roll-ups), even if it is just one or two puffs?’ and among those answering ‘yes’, those who indicated ‘I currently smoke cigarettes or hand-rolled cigarettes (roll-ups)’ were considered smokers. This does not include those who have smoked once or twice.

Family smoking (mother, father, (eldest) brother, (eldest) sister) was counted as the number of family members whom the student identified as a daily or occasional smoker. Responses ‘does not smoke’, ‘don’t know’, ‘do not have/see this person’, as well as non-responses were not counted as smokers. Family smoking was categorised into none, one, and two or more. Friends’ smoking was categorised into ‘none of them’, ‘some of them’, ‘about half of them’, ‘most of them’ and ‘don’t know’.

E-cigarette use was categorised as not having tried (coded 0), having tried once or twice (coded 1), or occasional or regular use (currently or any past regular use, coded 2). In 2013, there were no questions on e-cigarettes in the survey and all 2013 responses were therefore coded as 3.

Missing values for age (n=25) were imputed with the median age of their school year. For students with repeated measurements, missing observations of gender and ethnicity were imputed with values from previous or later surveys (n=82).

### Statistical analysis

The study population was described in terms of their sociodemographics, school year and smoking characteristics. Trends in tobacco accessibility, smoking norms and smoking attitudes over the survey years were graphically described and differences between survey years were tested using χ^2^ tests.

To study the association between time segments, and accessibility, norm and attitude outcomes we applied generalised estimating equations (GEE) analyses with a binomial distribution, logit link function, exchangeable correlation and robust standard errors. More information on the exact interpretation of the models can be found in the online Supplementary file. Stata V.15 was used for all analyses.

10.1136/tobaccocontrol-2018-054702.supp1 Supplementary data

Nested models were fitted, to first include sociodemographics, and then smoking-related variables. Model 1 included time segments, age, gender, FAS, school year and the school that the student was enrolled in. Model 2 additionally included smoking status, family smoking and friend smoking. As a post hoc analysis revealed a large confounding effect of e-cigarette use (see online supplementary table S1), we added e-cigarette use separately, only in Model 3.

10.1136/tobaccocontrol-2018-054702.supp2 Supplementary data

In order to establish whether potential reductions in accessibility, norm and attitude outcomes after the POS display ban are due to the ban, the associations between time segments and outcomes were studied by the level of frequency of shop visits. Differential associations were tested by assessing interaction between time and visits to shops (supermarkets and small shops separately) in the fully adjusted model.

As smoking can be a confounder as well as a mediator in the association, Model 1 may be underadjusted while Model 2 and Model 3 may be overadjusted. A sensitivity analysis was therefore conducted in which only never-smokers were included. As a second sensitivity analysis, we checked whether the missing data in 2013 affected the results of the change between 2014–2015 and 2016–2017, by excluding the 2013 data. Although not part of the initial data analysis plan, we performed a post hoc analysis on the baseline data (ie, only 2013) to assess whether interaction with shop visit frequency could have been expected, by examining the association between shop visit frequency and all three outcomes.

## Results

Table 1 describes the study sample in 2013, 2014–2015 and 2016–2017. E-cigarette use increased from 3% current/past occasional or regular e-cigarette users in 2014–2015 to 12% in 2016–2017 and the proportion of students reporting having no smokers in the family increased from 56% in 2013 to 65% in 2016–2017. The reported smoking behaviour of friends did not show a consistent decline, while the frequency of shop visits did not seem to change over time, with around 20% often visiting supermarkets, and around 40% often visiting small shops.

**Table 1:** Distribution of sociodemographic characteristics of the study population in all survey years, year before the partial ban (2013), years between the partial and comprehensive bans (2014–2015), and years after the comprehensive ban (2016–2017)

	All survey years	2013	2014–2015	2016–2017
N observations	6202	1357	2443	2402
Gender				
Male	51.1	51.7	51.4	50.4
Female	48.9	48.3	48.6	49.6
Age (mean, SD), years	14.5 (1.12)	14.6 (1.12)	14.5 (1.12)	14.5 (1.12)
12	<0.1	0	0	0.1
13	20.9	20.5	21.1	20.8
14	31.8	30.2	31.4	33.1
15	18.7	19.0	19.0	18.2
16	28.2	30.0	28.0	27.3
17	0.4	0.3	0.5	0.5
Ethnicity				
White	94.6	94.5	94.8	94.3
Non-white	5.4	5.5	5.2	5.7
School year				
Second year	53.0	51.1	52.8	54.2
Fourth year	47.0	48.9	47.2	45.8
Family Affluence Scale (FAS)				
Low	32.7	33.1	33.0	32.3
Intermediate	32.7	32.6	32.4	33.0
High	34.6	34.3	34.6	34.8
Smoking status				
Non-(current) smoker	95.4	94.7	96.0	95.3
Current smoker	4.6	5.3	4.0	4.7
E-cigarette use				
Never used	59.9	–	84.6	68.5
Used once or twice	12.5	–	12.1	20.0
Current/past occasional or regular use	5.8	–	3.3	11.5
2013 (missing)	21.9	100	–	–
Family smoking				
Non-smoking family members	61.2	56.0	60.1	65.3
One smoking family member	22.5	24.7	23.0	20.6
At least two smoking family members	16.3	19.3	16.8	14.1
Friend smoking				
None of them	54.1	55.1	60.3	47.3
At least some of them	34.2	34.3	28.0	40.5
Don’t know	11.6	10.5	11.7	12.2
Visits to supermarkets				
Rarely	21.4	20.0	21.4	22.3
Sometimes	57.0	56.4	56.2	58.1
Often	21.6	23.6	22.5	19.6
Visits to small shops				
Rarely	12.7	9.0	12.1	15.5
Sometimes	44.1	41.8	45.1	44.4
Often	43.2	49.2	42.8	40.1

Numbers represent percentages unless indicated otherwise.

**Figure 1:**
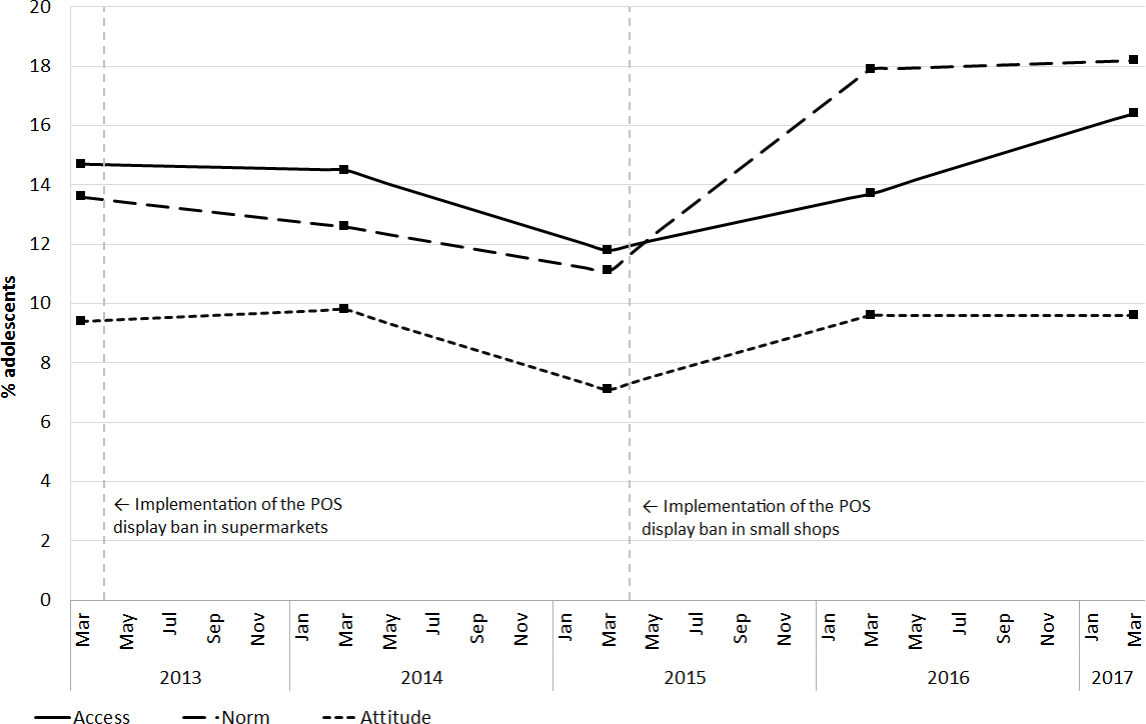
Crude trends in the percentage of adolescents who perceived tobacco to be purchasable in shops by someone their age (‘access’), who think their friends think it is OK for people their age to smoke (‘norm’), and who think it is OK for someone their age to smoke (‘attitude’). POS, point of sale.

Figure 1 presents the crude trends in perceived tobacco accessibility, smoking norm and smoking attitude. Prevalence decreased significantly between 2013 and 2015 for accessibility (14.7% to 11.8%, p=0.033) and positive smoking attitude (9.4% to 7.5%, p=0.032), while there was a non-significant decrease in the positive smoking norm (14.7% to 11.8%, p=0.052). Between 2015 and 2017 we found significant increases for accessibility (12.2% to 16.4%, p=0.001), smoking norm (11.3% to 18.2%, p<0.001) and smoking attitude (7.5% to 9.6%, p=0.021).

Table 2 presents the results of the GEE models. Model 1, adjusted for sociodemographics, did not show significant decreases over the time segments in odds of any of the three outcome measures. In Model 2, with smoking variables included, the changes over time remain very similar to those in Model 1. When additionally adjusted for e-cigarette use, in Model 3, the odds of tobacco accessibility decreased after the partial ban (OR=0.80, 95% CI 0.64 to 0.99) and comprehensive ban (OR=0.72, 95% CI 0.57 to 0.90) were implemented. Smoking attitude did not significantly change after the partial ban (OR=0.83, 95% CI 0.62 to 1.11), but odds of positive attitude were lower after the comprehensive ban was implemented (OR=0.67, 95% CI 0.49 to 0.91). Odds of a positive smoking norm did not change over time after controlling for all covariates (partial: OR=0.82, 95% CI 0.65 to 1.05; comprehensive: OR=1.00, 95% CI 0.78 to 1.29). Any previous occasional or regular e-cigarette use was associated with higher odds of perceived access (OR=3.12, 95% CI 2.32 to 4.21), positive norm (OR=2.94, 95% CI 2.16 to 4.02) and positive attitude (OR=3.38, 95% CI 2.35 to 4.87).

**Table 2:** ORs with 95% CIs from generalised estimating equation (GEE) models for three outcomes

	Tobacco accessibility*			Smoking norm†			Smoking attitude‡		
	Model 1	Model 2	Model 3	Model 1	Model 2	Model 3	Model 1	Model 2	Model 3
Time segments									
2013	Ref	Ref	Ref	Ref	Ref	Ref	Ref	Ref	Ref
2014–2015	0.90 (0.74 to 1.09)	0.97 (0.79 to 1.19)	0.80 (0.64 to 0.99)	0.86 (0.71 to 1.05)	1.05 (0.83 to 1.33)	0.82 (0.65 to 1.05)	0.89 (0.71 to 1.12)	1.10 (0.83 to 1.45)	0.83 (0.62 to 1.11)
2016–2017	1.11 (0.91 to 1.34)	1.04 (0.85 to 1.27)	0.72 (0.57 to 0.90)	1.54 (1.27 to 1.87)	1.53 (1.22 to 1.91)	1.00 (0.78 to 1.29)	1.09 (0.87 to 1.38)	1.09 (0.83 to 1.43)	0.67 (0.49 to 0.91)
School year, fourth versus second	3.06 (2.16 to 4.34)	2.41 (1.69 to 3.43)	2.38 (1.67 to 3.40)	4.07 (2.88 to 5.76)	2.76 (1.90 to 4.00)	2.76 (1.89 to 4.41)	4.00 (2.57 to 6.22)	2.71 (1.68 to 4.37)	2.73 (1.68 to 4.43)
Gender, female versus male	1.15 (0.99 to 1.33)	1.17 (1.00 to 1.37)	1.13 (0.97 to 1.32)	0.75 (0.65 to 0.88)	0.70 (0.59 to 0.83)	0.67 (0.56 to 0.79)	0.78 (0.65 to 0.94)	0.75 (0.61 to 0.93)	0.72 (0.58 to 0.89)
Age, per year increase	1.20 (1.03 to 1.39)	1.17 (1.00 to 1.36)	1.17 (1.00 to 1.36)	1.11 (0.96 to 1.30)	1.07 (0.90 to 1.26)	1.06 (0.90 to 1.26)	1.14 (0.95 to 1.37)	1.08 (0.87 to 1.32)	1.07 (0.87 to 1.32)
Ethnicity, non-white versus white	1.13 (0.81 to 1.56)	1.17 (0.84 to 1.62)	1.16 (0.84 to 1.61)	0.87 (0.61 to 1.24)	0.89 (0.60 to 1.33)	0.88 (0.59 to 1.32)	0.66 (0.42 to 1.05)	0.59 (0.37 to 0.94)	0.57 (0.35 to 0.93)
Family Affluence Scale (FAS)									
Low	Ref	Ref	Ref	Ref	Ref	Ref	Ref	Ref	Ref
Medium	1.15 (0.96 to 1.39)	1.21 (1.00 to 1.46)	1.17 (0.97 to 1.42)	1.10 (0.92 to 1.33)	1.25 (1.01 to 1.54)	1.21 (0.67 to 1.50)	0.89 (0.71 to 1.11)	1.02 (0.79 to 1.33)	0.99 (0.76 to 1.29)
High	1.18 (0.98 to 1.43)	1.35 (1.10 to 1.65)	1.30 (1.06 to 1.59)	0.90 (0.74 to 1.08)	1.20 (0.96 to 1.50)	1.16 (0.92 to 1.45)	0.78 (0.62 to 0.98)	1.15 (0.88 to 1.50)	1.09 (0.83 to 1.43)
Smoking status									
Non-(current) smoker		Ref	Ref		Ref	Ref		Ref	Ref
Current smoker		2.08 (1.58 to 2.75)	1.53 (1.14 to 2.05)		4.07 (3.07 to 5.39)	3.10 (2.31 to 4.16)		10.3 (7.57 to 14.0)	7.71 (5.61 to 10.6)
Family smoking									
None		Ref	Ref		Ref	Ref		Ref	Ref
One		0.95 (0.78 to 1.16)	0.88 (0.72 to 1.07)		1.33 (1.08 to 1.63)	1.21 (0.98 to 1.50)		1.47 (1.13 to 1.91)	1.34 (1.03 to 1.74)
At least two		1.29 (1.04 to 1.60)	1.18 (0.95 to 1.46)		1.53 (1.22 to 1.92)	1.37 (1.09 to 1.73)		2.19 (1.67 to 2.87)	1.90 (1.50 to 2.60)
Friend smoking									
None of them		Ref	Ref		Ref	Ref		Ref	Ref
At least some of them		3.08 (2.58 to 3.67)	2.62 (2.17 to 3.16)		12.0 (9.54 to 15.1)	10.3 (8.14 to 13.0)		6.71 (5.04 to 8.93)	5.67 (4.21 to 7.64)
Don’t know		1.00 (0.74 to 1.36)	1.00 (0.73 to 1.36)		1.81 (1.23 to 2.67)	1.81 (1.22 to 2.67)		2.29 (1.48 to 3.53)	2.30 (1.49 to 3.57)
E-cigarette use									
Never used			Ref			Ref			Ref
Used once or twice			1.66 (1.32 to 2.09)			1.77 (1.41 to 2.23)			1.69 (1.27 to 2.27)
Current/past occasional or regular use			3.12 (2.32 to 4.21)			2.94 (2.16 to 4.02)			3.38 (2.35 to 4.87)

*Tobacco accessibility was defined as perceiving tobacco to be purchasable in shops by someone their age.

†Smoking norm was defined as perceiving that friends think it is OK for people their age to smoke.

‡Smoking attitude was defined as thinking it is OK for someone their age to smoke.

Table 3 presents the results for the interactions between frequency of visiting supermarkets and time segments in the fully adjusted models. The changes in odds of outcomes over time were similar in the three levels of supermarket visit frequency. Table 4 presents the results for the interactions between frequency of visiting small shops and time segments. Changes in odds of positive smoking norms and attitudes over time were similar over the three levels of small shop visit frequency. Adolescents frequently visiting small shops showed a larger decline in odds of high perceived accessibility (comprehensive ban: OR=0.59, 95% CI 0.44 to 0.80, *p* for interaction =0.008) than those visiting rarely (OR=2.42, 95% CI 0.89 to 6.62). Findings for smoking norms and attitudes did not vary by shop visit frequency.

**Table 3:** ORs with 95% CIs for three outcomes in three groups of frequency of visits to supermarkets, as derived from generalised estimating equation (GEE) models that included interaction between time segments and frequency of visits to supermarkets. Interactions test differential associations between time segments and outcomes, between exposure groups

	Frequency of visits to supermarkets				
	Rarely	Sometimes	P for interaction*	Often	P for interaction*
**Tobacco accessibility†**					
Time segments					
2013	Ref	Ref		Ref	
2014–2015	0.75 (0.50 to 1.13)	0.93 (0.69 to 1.24)	0.404	0.63 (0.43 to 0.94)	0.563
2016–2017	0.68 (0.44 to 1.03)	0.84 (0.62 to 1.13)	0.383	0.58 (0.37 to 0.87)	0.566
**Smoking norm‡**					
Time segments					
2013	Ref	Ref		Ref	
2014–2015	0.84 (0.53 to 1.35)	0.83 (0.59 to 1.17)	0.975	0.74 (0.48 to 1.15)	0.684
2016–2017	0.87 (0.54 to 1.38)	1.13 (0.82 to 1.59)	0.329	0.90 (0.57 to 1.42)	0.910
**Smoking attitude§**					
Time segments					
2013	Ref	Ref		Ref	
2014–2015	0.95 (0.53 to 1.72)	0.79 (0.53 to 1.18)	0.613	0.81 (0.48 to 1.39)	0.694
2016–2017	0.88 (0.49 to 1.59)	0.64 (0.42 to 0.96)	0.338	0.62 (0.36 to 1.08)	0.378

*Rarely was the reference category in the interaction analysis.

†Tobacco accessibility was defined as perceiving tobacco to be purchasable in shops by someone their age.

‡Smoking norm was defined as perceiving that friends think it is OK for people their age to smoke.

§Smoking attitude was defined as thinking it is OK for someone their age to smoke.

**Table 4:** ORs with 95% CIs for three outcomes in three groups of frequency of visits to small shops, as derived from generalised estimating equation (GEE) models that included interaction between time segments and frequency of visits to small shops. Interactions test differential associations between time segments and outcomes, between exposure groups

	Frequency of visits to small shops				
	Rarely	Sometimes	P for interaction*	Often	P for interaction*
**Tobacco accessibility†**					
Time segments					
2013	Ref	Ref		Ref	
2014–2015	2.55 (0.91 to 7.14)	1.09 (0.77 to 1.55)	0.126	0.62 (0.47 to 0.82)	0.010
2016–2017	2.42 (0.89 to 6.62)	0.93 (0.65 to 1.33)	0.077	0.59 (0.44 to 0.80)	0.008
**Smoking norm‡**					
Time segments					
2013	Ref	Ref		Ref	
2014–2015	0.51 (0.22 to 1.17)	0.83 (0.56 to 1.23)	0.302	0.91 (0.66 to 1.24)	0.202
2016–2017	0.66 (0.32 to 1.39)	1.29 (0.88 to 1.88)	0.112	0.97 (0.69 to 1.36)	0.344
**Smoking attitude§**					
Time segments					
2013	Ref	Ref		Ref	
2014–2015	0.69 (0.26 to 1.87)	0.89 (0.55 to 1.44)	0.650	0.85 (0.59 to 1.24)	0.697
2016–2017	0.43 (0.17 to 1.08)	0.67 (0.41 to 1.09)	0.403	0.79 (0.53 to 1.17)	0.233

*Rarely was the reference category in the interaction analysis.

†Tobacco accessibility was defined as perceiving tobacco to be purchasable in shops by someone their age.

‡Smoking norm was defined as perceiving that friends think it is OK for people their age to smoke.

§Smoking attitude was defined as thinking it is OK for someone their age to smoke.

Sensitivity analyses are presented in online supplementary tables S2 and S3. Online supplementary table S2 shows that the declines in odds of high perceived accessibility and positive attitudes observed for the total population were smaller and non-significant in the subset of never-smokers (OR=0.97, 95% CI 0.73 to 1.29 and OR=0.87, 95% CI 0.53 to 1.40, respectively). Table S3 demonstrates that the changes observed between 2014–2015 and 2016–2017 were practically identical when 2013 data were included or excluded. Given that we only found tobacco accessibility to be associated with small shop visit frequency at baseline (see online supplementary table S4), interaction with supermarket visits was, in hindsight, unexpected for all three outcomes.

## Discussion

### Key findings

The perceived accessibility of tobacco and positive smoking norm and attitude among Scottish adolescents appears to have increased over time in the crude data. However, when controlled for all covariates, including e-cigarette use, the implementation of partial and comprehensive POS display bans was followed by a decrease in perceived tobacco accessibility and a more negative attitude towards smoking. Smoking norm did not significantly change when fully adjusted. Adolescents who more frequently visited small shops showed the largest reductions in perceived accessibility, but we did not find the change in smoking norms and attitudes to vary by shop visit frequency.

### Limitations

This study used a strong study design, large sample with high response rates and 5 years of data which included both partial and comprehensive implementation of the POS display ban. However, these results need to be interpreted in light of some limitations.

First, all variables used in this study were self-reported. The surveys were anonymous and all surveys were conducted in schools, which has been shown to result in limited bias.^24 25^ Nevertheless, because we measured changes in smoking norms and attitudes, there is a risk of desirability bias in young people’s responses. If the introduction of a POS display ban caused individuals to provide more socially desirable answers (ie, less accepting of smoking) but not change their personal opinion, this may have caused an overestimation of the association.

Second, participants attended a limited number of schools in Scotland and therefore may not be representative of the Scottish school population. However, comparison of the smoking characteristics of the DISPLAY Study in 2013 with a nationally representative data of the 2013 SALSUS sample did not indicate any significant deviation on never-smoking and smoking attitude.^26^

Third, we acknowledge two limitations in the measurement of e-cigarette use. E-cigarette use was not measured in 2013, because it was not considered a relevant problem among Scottish youth due to the very low prevalence at that time. Additionally, we could not distinguish between current and past occasional or regular use, which may have diluted the association between e-cigarette use and outcomes. Therefore some confounding by e-cigarettes use could not be taken into account.

### Interpretation

#### Attributability to the POS display ban

We found a decline in perceived tobacco accessibility and a more negative attitude towards smoking after the implementation of POS display bans, compared with preban. The data used were part longitudinal, and our findings are consistent with other studies that found decreases in perceived tobacco access and smoking acceptability following POS display bans.^14 15 19^ It is however important to evaluate in detail whether the observed changes are causally attributable to the POS display ban. In the paragraphs below we discuss how four issues are unlikely to have led to impaired causal inference.

First, we believe that other tobacco control measures over the same period are unlikely to have contributed substantially to the changes observed. Tobacco taxes increased each year, but not by a larger amount in 2013 and 2015.^27^ Regulations mandating the standardised packaging of cigarettes and a ban on packs containing less than 20 sticks came into force in the UK in May 2016, but these changes were not substantially implemented until the last few months of the 12-month transition period (ie, February to May 2017)^28^ and therefore were not fully in place during the last wave of data collection. We therefore consider it unlikely that the regulations had a significant impact on adolescents’ perceptions of access, norms and attitude in the period studied. The minimum age on tobacco sales was 18 years throughout the study period,^29^ but there were two mass media campaigns in Scotland that addressed tobacco accessibility. However, these campaigns targeted adults, helping tobacco retailers adhere to age verification regulation and warning those aged 18+ years not to buy cigarettes for minors. Although these may have some effect on perceptions of young people, we consider it unlikely that a substantial part of our findings can be explained by these campaigns.

Second, we did not consistently find a stronger association between the display ban and the outcomes among adolescents who were more frequently exposed to retailers. However, this ‘dose-response effect’^30^ could not have been reasonably expected, as there was a lack of association at baseline (see online supplementary table S2). An effect of POS display bans on adolescents’ perceived acceptability of smoking may not depend on individual exposure, as social norms are transferred within wide social networks.^31^ The distinction between levels of exposure was therefore less meaningful than expected, but does not per se disprove causality.

Third, even though we were unable to take longer-term trends before and after the implementation of POS display bans into account, previous studies support effects over and above secular trends. A study that evaluated the POS display ban among adults in England controlled for the preban secular trend and found that the month-by-month trend in smoking declined more rapidly after the introduction of the partial display ban.^32^ Moreover, an international comparative study found that the decline in adolescent smoking prevalence in countries that implemented POS display bans was larger than the secular decline in countries that did not.^17^

Lastly, we found some inconsistency in results for different outcomes (attitudes towards smoking became more negative, but the smoking norm did not change), but this may be explained by the timing of changes. Injunctive norms (ie, perceiving that others accept smoking) may take longer to be perceived and reported, if opinions of peers need to have been exchanged and established before individuals perceive these as the norm. Descriptive norms (ie, perceiving a high smoking prevalence) may change quicker as it involves perception of directly observable behaviour. Previous studies observed a more short-term change in descriptive norms.^14 15 19^ As descriptive and injunctive norms interact in influencing individual behaviour,^33^ effects may be detected in the long term.

Overall, we conclude that the associations observed may be causal. We do acknowledge that further studies are needed that take some of the discussed issues into account, within the possibilities of real world settings.

#### Role of e-cigarettes

The crude trends showed an increase in tobacco accessibility and positive smoking attitude between 2015 and 2017, but after adjustment for e-cigarette use in the model, odds decreased between 2013 and 2016–2017. We found positive associations between e-cigarette use and our three outcomes (table 2) and that e-cigarette use increased over time (table 1). The latter finding can indicate a co-occurring, but unrelated, trend with the implementation of the display ban or it may indicate that the display ban unintentionally aided in increasing e-cigarette use. In both scenarios, our findings suggest that part of the crude positive trends are attributable to an increasing trend in e-cigarette use, and that e-cigarette use may have inhibited de-normalisation of smoking and, therefore could have potentially reduced some of the intended impact of the POS display ban.

The debate about whether e-cigarettes can renormalise tobacco smoking is ongoing.^34–37^ Renormalisation may be driven by the considerable conceptual overlap between e-cigarettes with conventional cigarettes, such as the similarity in smoking and vaping rituals, visual similarity, cultural overlaps in user groups, and similarity in advertising at POS prior to the display ban.^38^ The renormalisation hypothesis is supported by the current study. A study from the UK showed that perceived harm of smoking reduced when adolescents were exposed to e-cigarette advertising,^39^ and a US study demonstrated that adolescent never-smokers were more accepting of adult smoking when they used e-cigarettes, were exposed to e-cigarette advertising or lived with e-cigarette users.^40^

Although there is some evidence for a renormalising effect, e-cigarettes may primarily be considered an alternative to conventional cigarettes.^38^ E-cigarettes may be considered attractive in part due to their limited health risks compared with conventional cigarettes, which emphasises that the high risks of smoking are not accepted and that the normalisation of e-cigarettes can progress without obstructing the continued denormalisation of tobacco.^38^ A Scottish qualitative study, funded by an e-cigarette company, suggested that e-cigarettes were perceived as a smoking cessation aid, and that adolescents were not more attracted to tobacco smoking when seeing e-cigarettes being used.^41^

The UK shows a population-wide increasing trend in the use of e-cigarettes in adults^42^ as well as adolescents.^43^ Further independent empirical studies are needed to establish the role of e-cigarettes in perceptions of smoking norms among adolescents. A fundamental question in such future studies would be to what extent adolescent survey respondents distinguish the terminology for and connotations of e-cigarette use and tobacco smoking.

#### Policy implications

We found that tobacco accessibility declined after the implementation of the partial POS display ban (in supermarkets), but only the comprehensive ban (in all shops) significantly reduced positive smoking attitudes. A 2013 (predisplay ban) study in Scotland showed that both for supermarkets and small shops 80% of adolescents recalled seeing tobacco products or promotions.^12^ Only banning displays in supermarkets is therefore not sufficient and our findings support the need for comprehensive bans on tobacco display in countries where tobacco is still visible at POS.

## Conclusions

The ban on the open display of tobacco products in supermarkets and small shops in Scotland was followed by a reduction in adolescents’ perception of the accessibility of tobacco and their positive attitudes towards smoking, but only when taking the increase in e-cigarette use over 2013–2017 into account. The role of e-cigarettes in the perception of smoking acceptability is a topic of further study.

**What this paper adds**

**What is already known on this subject**

- Tobacco displays at the point-of-sale (POS) remain an important marketing tool for the tobacco industry to target young people.
- Among Scottish adolescents, 80% reported having seen tobacco displays before implementation of a POS tobacco display ban and the impact of such a ban is therefore potentially substantial.

**What important gaps in knowledge exist on this topic**

- Few studies to date have evaluated the impact of removing POS tobacco displays on adolescents, and existing studies have not assessed changes in adolescents’ perceptions of the acceptability of smoking.
- Scotland implemented a display ban in two phases in 2013 and 2015, and this natural experiment provides important evidence for other countries on the importance of display bans for tobacco control in general, and youth smoking prevention in particular.

**What this paper adds**

- The POS tobacco display ban in supermarkets and small shops in Scotland was followed by reductions in adolescents’ perceived accessibility of tobacco and positive attitude towards smoking, but only after taking into account that the use of e-cigarettes increased between 2013 and 2017.
