# Supplementary material for: Adolescents’ perceptions of tobacco accessibility and smoking norms and attitudes in response to the tobacco point-of-sale display ban in Scotland: results from the DISPLAY Study
Source: Tob Control. 2019 May 3;29(3):348–56. doi: 10.1136/tobaccocontrol-2018-054702 (PMC7231455; doi:10.1136/tobaccocontrol-2018-054702)
Supplement: Supplementary data [file tobaccocontrol-2018-054702supp001.pdf]

SUPPLEMENTARY FILE

In a Generalised Estimating Equations (GEE) analysis the independent variables can be interpreted in the same way as for any generalized linear model estimating a population averaged effect. For example in the basic model with the binary dependent variable 'accessibility' if we add only the independent variable for 'year' i.e. 2013, 2014-2015 and 2016-2017 (with reference category 2013), then the estimated odds ratios for 2014-2015 and 2016-2017 quantify the difference in odds of perceiving tobacco as accessible between these years relative to 2013 (on a multiplicative scale e.g. three times the odds or half the odds).

When e-cigarette ever-use is added into the model the odds ratio for e-cigarettes can be interpreted as how much higher the odds are of perceiving tobacco as accessible in young people who have used e-cigs compared to those who have not (in the fully adjusted models we found an odds ratio of 3). The effect is estimated as constant across all the years we have e-cigarette data for. With the e-cigarette variable in the model the interpretation of the odds ratios for 2014-2015 and 2016-2017 becomes 'how much higher the odds are of a never e-cig user (the reference category) in 2014-2015 perceiving tobacco as accessible compared to the odds for all the young people in 2013' (all in 2013 because we can't adjust for e-cig use in 2013).

It is unfortunate that we do not have data for e-cigarette use in 2013, however, the reason for this is that e-cig use is a relatively recent phenomenon; use only really began around 2012 and was still very low level in 2013. SALSUS data indicate that 88% of this age group in Scotland had never tried an e-cig in 2013. Therefore the odds in all participants in 2013 will closely approximate the odds for e-cigarette never users in 2013.

The model estimation process (by quasi maximum likelihood) for GEE finds the coefficients that make obtaining the observed data most likely. In this case, the model's results indicate that e-cigarette users have significantly greater odds of perceiving tobacco as accessible and of having positive smoking norms and attitudes (note that causality cannot be established in these associations).
