# Supplementary material for: Adolescents’ perceptions of tobacco accessibility and smoking norms and attitudes in response to the tobacco point-of-sale display ban in Scotland: results from the DISPLAY Study
Source: Tob Control. 2019 May 3;29(3):348–56. doi: 10.1136/tobaccocontrol-2018-054702 (PMC7231455; doi:10.1136/tobaccocontrol-2018-054702)
Supplement: Supplementary data [file tobaccocontrol-2018-054702supp002.pdf]

SUPPLEMENTARY TABLES

Table S1: Post-hoc analysis: the confounding effect of e-cigarette use in the trend in outcomes over time.

	**Access**	**Norm**	**Attitude**
**Model A:** year segments, e-cigarettes			
Time segments			
2013	Ref	Ref	Ref
2014-2015	0.65 (0.54-0.80)	0.54 (0.44-0.67)	0.51 (0.40-0.66)
2016-2017	0.56 (0.45-0.69)	0.59 (0.48-0.74)	0.34 (0.26-0.46)
E-cigarette use			
Never used	Ref	Ref	Ref
Used once or twice	3.04 (2.48-3.73)	4.69 (3.85-5.71)	4.97 (3.88-6.37)
Current/past occasional or regular use	7.25 (5.65-9.29)	12.1 (9.48-15.5)	16.8 (12.7-22.2)
**Model B:** year segments, school, school year, gender, age, ethnicity, FAS, smoking, family smoking, e-cig. use			
Time segments			
2013	Ref	Ref	Ref
2014-2015	0.72 (0.59-0.88)	0.64 (0.51-0.79)	0.68 (0.52-0.90)
2016-2017	0.70 (0.56-0.87)	0.90 (0.72-1.14)	0.62 (0.46-0.85)
E-cigarette use			
Never used	Ref	Ref	Ref
Used once or twice	2.15 (1.72-2.68)	2.94 (2.37-3.64)	2.46 (1.86-3.27)
Current/past occasional or regular use	4.49 (3.36-6.00)	6.09 (4.48-8.27)	5.62 (3.89-8.13)
**Model C:** year segments, school, school year, gender, age, ethnicity, FAS, e-cigarette use			
Time segments			
2013	Ref	Ref	Ref
2014-2015	0.69 (0.57-0.85)	0.56 (0.45-0.69)	0.54 (0.41-0.69)
2016-2017	0.64 (0.51-0.79)	0.69 (0.55-0.87)	0.39 (0.29-0.52)
E-cigarette use			
Never used	Ref	Ref	Ref
Used once or twice	2.36 (1.91-2.93)	3.74 (3.04-4.60)	3.82 (2.94-4.95)
Current/past occasional or regular use	5.89 (4.52-7.67)	11.1 (8.49-14.5)	14.9 (11.0-20.2)
**Model D:** year segments, school, school year, gender, age, ethnicity, FAS, friends smoking			
Time segments			
2013	Ref	Ref	Ref
2014-2015	0.97 (0.89-1.18)	1.02 (0.82-1.28)	1.04 (0.81-1.34)
2016-2017	1.01 (0.82-1.23)	1.40 (1.13-1.73)	0.95 (0.75-1.22)
Friend smoking			
None of them	Ref	Ref	Ref
At least some of them	3.51 (2.97-4.16)	15.3 (12.2-19.1)	11.2 (8.52-14.7)
Don’t know	1.02 (0.75-1.38)	1.90 (1.29-2.80)	2.52 (1.64-3.87)

FAS = Family Affluence Scale
E-cig = e-cigarette

Table S2: Sensitivity analysis 1: analysis conducted in only never-smokers (5,014 observations; 81% of sample). This model excludes smoking status (as there are no smokers), but adds smoking susceptibility.

	Tobacco accessibility[a]	Smoking norm[b]	Smoking attitude[c]
Time segments			
2013	Ref	Ref	Ref
2014-2015	1.00 (0.76-1.31)	1.18 (0.83-1.69)	1.26 (0.79-2.00)
2016-2017	0.97 (0.73-1.29)	1.62 (1.14-2.31)	0.82 (0.49-1.37)
School year, 4th year vs 2nd year	2.37 (1.53-3.68)	2.82 (1.68-4.74)	5.57 (2.54-12.2)
Gender, female vs male	1.25 (1.02-1.53)	0.84 (0.66-1.06)	0.89 (0.63-1.25)
Age, per year increase	1.14 (0.94-1.38)	1.05 (0.83-1.32)	0.84 (0.60-1.18)
Ethnicity, non-white vs white	1.10 (0.71-1.70)	1.12 (0.66-1.87)	0.56 (0.20-1.56)
Family affluence (FAS)			
Low	Ref	Ref	Ref
Medium	1.25 (0.97-1.61)	1.00 (0.74-1.34)	1.00 (0.66-1.52)
High	1.35 (1.03-1.76)	0.96 (0.70-1.32)	0.94 (0.59-1.47)
E-cigarette use			
Never used	Ref	Ref	Ref
Used once or twice	1.38 (1.00-1.90)	0.99 (0.70-1.40)	0.98 (0.60-1.60)
Occasional or regular	2.71 (1.56-4.72)	1.37 (0.73-2.56)	1.98 (0.87-4.50)
Smoking susceptibility[d]			
Non-susceptible	Ref	Ref	Ref
Susceptible	1.56 (1.26-1.94)	1.74 (1.34-2.24)	3.54 (2.43-5.15)
Family smoking			
0	Ref	Ref	Ref
1	0.88 (0.68-1.15)	1.28 (0.96-1.72)	1.79 (1.17-2.73)
≥2	1.23 (0.93-1.64)	1.24 (0.88-1.74)	2.48 (1.56-3.92)
Friend smoking			
None of them	Ref	Ref	Ref
At least some	2.49 (1.99-3.11)	8.70 (6.51-11.6)	4.02 (2.65-6.10)
Don't know	1.03 (0.73-1.44)	1.48 (0.91-2.39)	2.17 (1.25-3.77)

[a] Tobacco accessibility was defined as perceiving tobacco to be purchasable in shops by someone their age.
[b] Smoking norm was defined as perceiving that friends think it is OK for people their age to smoke.
[c] Smoking attitude was defined as thinking it is OK for someone their age to smoke.
[d] This model excludes smoking status (as there are no smokers), but adds smoking susceptibility.

Table S3: Sensitivity analysis 2: quantifying the change between 2014-2015 and 2016-2017, excluding the 2013 data. Presented analyses were adjusted for age, gender, ethnicity, FAS, school year, school, smoking status, e-cigarette use, family smoking, and friend smoking.

	Tobacco accessibility[a]		Smoking norm[b]		Smoking attitude[c]	
	All years	2013 excluded	All years	2013 excluded	All years	2013 excluded
Time segments						
2014-2015	Ref	Ref	Ref	Ref	Ref	Ref
2013	1.25 (1.01-1.54)	-	1.22 (0.96-1.55)	-	1.20 (0.89-1.61)	-
2016-2017	0.90 (0.75-1.08)	0.89 (0.74-1.07)	1.22 (1.00-1.49)	1.20 (0.98-1.46)	0.80 (0.62-1.03)	0.79 (0.61-1.02)

[a] Tobacco accessibility was defined as perceiving tobacco to be purchasable in shops by someone their age.
[b] Smoking norm was defined as perceiving that friends think it is OK for people their age to smoke.
[c] Smoking attitude was defined as thinking it is OK for someone their age to smoke.

Table S4: Post-hoc analysis: check whether there is an association between frequency of shop visits and three outcomes, before the display bans were implemented (2013). Logistic regression analysis controlled for school year, gender, age, ethnicity, FAS, school, smoking status, e-cig use, family smoking, and friend smoking.

	**Odds ratio (95% confidence interval)**	
	**Supermarket visits**	**Small shop visits**
**Tobacco accessibility[a]**		
Rarely	Ref	Ref
Sometimes	0.72 (0.47-1.08)	2.68 (1.03-6.99)
Often	1.42 (0.88-2.27)	5.64 (2.20-14.5)
**Smoking norm[b]**		
Rarely	Ref	Ref
Sometimes	0.68 (0.42-1.08)	0.79 (0.39-1.60)
Often	0.98 (0.57-1.69)	1.19 (0.60-2.38)
**Smoking attitude[c]**		
Rarely	Ref	Ref
Sometimes	1.12 (0.64-1.99)	0.94 (0.39-2.22)
Often	1.48 (0.77-2.83)	1.27 (0.55-2.93)

[a] Tobacco accessibility was defined as perceiving tobacco to be purchasable in shops by someone their age.
[b] Smoking norm was defined as perceiving that friends think it is OK for people their age to smoke.
[c] Smoking attitude was defined as thinking it is OK for someone their age to smoke.
